# Longitudinal change in restricted and repetitive behaviors from 8-36 months

**DOI:** 10.1186/s11689-020-09335-0

**Published:** 2021-01-11

**Authors:** Robin Sifre, Daniel Berry, Jason J. Wolff, Jed T. Elison

**Affiliations:** 1 Institute of Child Development, University of Minnesota, 51 East River Parkway, Minneapolis, MN 55455 USA; 2 Department of Educational Psychology, University of Minnesota, Minneapolis, MN USA

## Abstract

**Background:**

Restricted and repetitive behaviors (RRBs) are core features of autism spectrum disorder (ASD) and one of the earliest behavioral signs of ASD. However, RRBs are also present in typically developing (TD) infants, toddlers, and preschool-aged children. Past work suggests that examining change in these behaviors over time is essential to distinguish between normative manifestations of these behaviors and behaviors that denote risk for a neurodevelopmental disorder. One challenge in examining changes in these behaviors over time is that most measures of RRBs have not established longitudinal measurement invariance. The aims of this study were to (1) establish measurement invariance in the Repetitive Behavior Scales for Early Childhood (RBS-EC), a parent-report questionnaire of RRBs, and (2) model developmental change in RRBs from 8 to 36 months.

**Methods:**

We collected RBS-EC responses from parents of TD infants (*n* = 180) from 8 to 36 months (*n* = 606 responses, with participants contributing an average of 3-time points). We leverage a novel methodological approach to measurement invariance testing (Bauer, Psychological Models, 22(3), 507–526, 2017), moderated nonlinear factor analysis (MNLFA), to determine whether the RBS-EC was invariant across age and sex. We then generated adjusted factor score estimates for each subscale of the RBS-EC (repetitive motor, self-directed, and higher-order behaviors), and used linear mixed effects models to estimate between- and within-person changes in the RBS-EC over time.

**Results:**

The RBS-EC showed some non-invariance as a function of age. We were able to adjust for this non-invariance in order to more accurately model changes in the RBS-EC over time. Repetitive motor and self-directed behaviors showed a linear decline from 8 to 36 months, while higher-order behaviors showed a quadratic trajectory such that they began to decline later in development at around 18 months. Using adjusted factor scores as opposed to unadjusted raw mean scores provided a number of benefits, including increased within-person variability and precision.

**Conclusions:**

The RBS-EC is sensitive enough to measure the presence of RRBs in a TD sample, as well as their decline with age. Using factor score estimates of each subscale adjusted for non-invariance allowed us to more precisely estimate change in these behaviors over time.

**Supplementary Information:**

The online version contains supplementary material available at 10.1186/s11689-020-09335-0.

## Introduction

### Restricted and repetitive behaviors

The DSM-5 defines autism as a disorder marked by social deficits, as well as “restricted, repetitive patterns of behavior, interests, or activities” [1]. These restricted and repetitive behaviors (RRBs) include repetitive motor movements, ritualistic behaviors, repetitive self-injury, inflexibility, and circumscribed and intense interests. RRBs are among the earliest detectable behavioral markers of ASD [43], with evidence from direct observation indicating that elevated frequencies of RRBs in children with ASD can be identified as early as 12 months [16, 22, 43]. Parent report of RRBs, as measured by the Repetitive Behavior Scale-Revised (RBS-R [7]), indicates that 12-month-old infants at high-familial risk for ASD who later received a diagnosis (HR-ASD) have elevated repetitive behaviors relative to high-risk infants who do not receive an ASD diagnosis (HR-Neg) and low-risk children [55], raising the possibility of using parent reports of RRBs as an inexpensive early screening tool for ASD.

One challenge with characterizing RRBs, and especially early in development, is that they are not specific to ASD. Rather, they manifest in samples of typically developing children [3, 15, 18, 20, 31, 49, 50, 56] and across a range of neurodevelopmental disorders and monogenic syndromes [17, 23, 33, 39, 54]. Because these behaviors variably manifest across age and across the typical-to-atypical continuum [10], quantifying variability in selected topographies requires consideration of normative patterns of development and establishing psychometric integrity on samples that include children who are typically developing. While some behaviors may be specific to ASD at certain ages and levels of functioning (e.g., lateral glances and/or unusual visual inspection in a 10-year-old), most topographies of repetitive behavior are observed in typically developing children at some point over the course of development. Therefore, establishing the psychometric integrity of measures that can capture meaningful variability across the typical-to-atypical continuum is essential to advance our understanding of the constellation of features that defines ASD [11, 12].

Further, to distinguish between normative RRBs and those that are predictive of risk for an emerging neurodevelopmental disorder, it is critical to look at change in RRBs over time. One study, Uljarević et al. [53], examined longitudinal change in RRBs in TD children as measured through parent report (the Repetitive Behavior Questionnaire-2; RBQ-2 [31]) and found that lower-order behaviors (e.g., motor stereotypies) decreased from 15 to 77 months, whereas higher-order behaviors (e.g., insistence on sameness) peaked around 26 months of age before declining. These findings suggest that while the presence of RRBs early in development may not be atypical, their persistence over time may be.

In order to effectively measure changes in RRBs across development, it is imperative that investigators are confident that their measures reflect the same underlying construct whether parents are reporting on a 9-month-old or a 36-month-old. One previous study examining change in two RRB sub-types showed that the underlying two-factor structure was stable across 3-time points (15, 26, and 77 months), suggesting that the underlying structure of the RBQ-2 was stable across development [53]. Referred to as “configural invariance” in the psychometrics literature, establishing a common factor structure over time is a crucial first step to assuring the developmental interpretability of a measure. That is, if the nature of the underlying construct changes qualitatively over time, then quantitative estimates of substantive developmental change (i.e., apples to apples) become inextricably confounded with changes in the meaning of the measure (i.e., apples to oranges). However, as is discussed under the broader heading of ‘measurement invariance’ (MI) testing, configural invariance is a necessary—though not sufficient—step to assuring meaningful comparisons across groups or within-individuals over time.

### What is measurement invariance and why should we care?

MI for a scale exists when “a scale or construct provides the same results across several different samples or populations” (APA 2014 [2], p. 211; see also [44]). In other words, a scale is invariant if the distribution of responses obtained from a group of individuals depends only on their responses which the measurement is intended to reflect, and not on other demographic characteristics such as age or diagnostic group [36]. Without MI, one cannot validly compare scores on a given scale between groups or within individuals over time. Differential item functioning (DIF) between male and female respondents on measures of depression is a prototypic example. Due to social norms, girls may tend to endorse the item “cries easily” more often than do boys [47], leading to inflated estimated depression scores for girls because of item bias. To accurately compare rates of depression between boys and girls, one must account for items that function differently due to sex as opposed to true differences in the underlying construct of interest (depression).

Similarly, items on any questionnaire measuring RRBs may function differently at different ages. Thus, to model substantively meaningful change in the underlying constructs, we must first evaluate whether the measure is invariant across time. Though this issue has not yet been explicitly discussed in the context of RRBs, researchers have noted how challenging it is to find a measure that is sensitive to both age and cognitive development and differences across diagnostic groups [24]. Indeed, the notable variability in factor solutions that have been proposed in the literature is likely due to both (1) substantively meaningful differences in RRBs across the populations and ages studied, and (2) measurement sensitivity differences due to age and diagnostic status. In order to distinguish between these two sources of variability, it is critical that the field test MI and correct for sources of non-invariance.

Factor analysis commonly considers three degrees of MI. A measure has *configural invariance* when it has the same factor structure across time (e.g., items load on to the same latent factors across time) [41]. Researchers must also test for the possibility that a latent factor *means* something slightly different across groups or ages. This is typically referred to as *metric* non-invariance, and indicates that there are age differences in the factor loadings (or that the items are not reflecting the same construct over time). For example, in typically developing infants, the relation between a latent construct like *repetitive motor behaviors* and the item “repeatedly mouths objects” (i.e., the factor loading) may differ between young infants and 4-year-olds because repetitive mouthing may be more meaningful for the latent construct in infancy, when mouthing is developmentally normative. By failing to account explicitly for these differences in the measurement model, we would be essentially comparing apples and oranges.

Assuming invariant factor loadings across groups (metric invariance), it remains possible that, despite showing identical levels of *latent* repetitive behaviors in infancy and at age four, the same “repetitive mouthing” item may be rated as higher in infancy, on average, because of normative developmental differences in object exploration. This is typically referred to as *scalar* non-invariance. This would mean that—without explicit adjustment in the measurement model—this difference would sneak into our estimate of the construct mean and lead to the spurious conclusion that repetitive behavior decreases between infancy and early childhood. Adjusting for these biases is crucial for any meaningful interpretation of development.

Traditionally, applied researchers have leveraged two approaches for measuring and adjusting MI: multiple groups analysis (MGA [25]) and multiple indicators, multiple causes (MIMIC) modeling [26]. Each has specific strengths and weaknesses. MGA is perhaps the most commonly used approach in MI testing and is especially useful when one is concerned about MI across discrete groupings of individuals (e.g., sex, race). Briefly, MGA works by fitting taxonomies of confirmatory factor analytic models (CFAs) jointly to multiple covariance matrices—one for each group—and systematically testing the extent to which the parameters of interest (e.g., factor loadings, indicator intercepts, indicator residual (co)variances, factor (co)variances) can be constrained to equality across groups without significantly undermining overall model fit (i.e., aggregate model fit across groups). To the extent to which the parameters can be constrained to equality, the latent variables are said to be invariant. In cases in which some (but not all) of the parameters can be constrained to equality, one invokes ‘partial’ MI [8]. Although there is debate about just *how* non-invariant groups can be before the substantive meaning of the construct differs across groups (see [9]), the key strength of testing MI is that it allows one to quarantine these differences to the measurement model. This approach essentially removes sources of potential bias from the latent construct and subsequent substantive analyses.

A core advantage to MGA is that it allows one to model heterogeneity in all of the parameters of typical psychometric interest (e.g., factor loadings; item intercepts; factor (co)variances, item residual (co)variances). Specifically, MGA allows one to test and adjust for the possibility of both metric and scalar invariance. MGA can be a powerful tool toward this end. However, it also has some non-trivial weaknesses. MGA becomes intractable quickly, as the number of discrete groupings extends beyond a few categories. Also, in order to apply MGA to continuous moderators, such as age or income, one has to discretize continuously distributed scales in typically arbitrary ways.

**Fig. 1:**
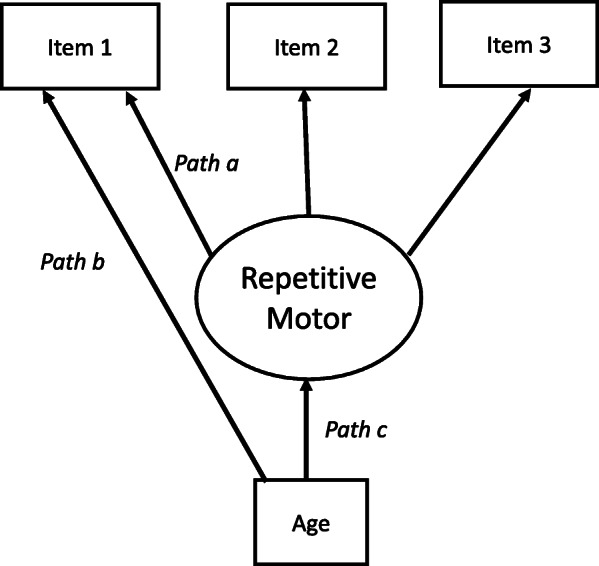
Example of MIMIC model. An individual’s response on item 1 is due to the underlying latent construct of repetitive motor behaviors (path a) as well as an age moderator (path b). Path c represents the factor loading on item 1 after adjusting for the moderator

MIMIC models take a slightly different approach to MI testing that can help to address some of these weaknesses. Specifically, rather than fitting simultaneous measurement models to separate covariance matrices and testing systematic equality constraints across the discrete groups, MIMIC models address MI in a manner akin to regression adjustment (Fig. 1). Here, an individual’s level on a given indicator (e.g., repeated mouthing of object) is considered to be due to the underlying latent construct (i.e., repetitive behavior) (path a), as well as one or more continuous (e.g., age) or categorical (e.g., sex) moderators (path b). Similar to multiple regression, by simultaneously regressing, the indicator and the latent construct on the moderator(s), the factor loading to the indicator (path b) would represent the unique relation between the construct and the indictor that remains—*after adjusting for the moderator* (path c). It could also be considered a variant of a mediational model, such that the moderator is thought to have a direct effect on the indicator, as well as an indirect effect on the indicator by virtue of its effect on the latent construct (paths a×b). In principle, as long as the latent variable model is identified, one could include any number or predictors, along with any potentially meaningful interactions between them—an advantage over MGA. A notable downside to traditional MIMIC models, however, is that they typically adjust only for scalar invariance. Modeling systematic difference in any of the other parameters of typical interest (i.e., factor loadings, factor, and/or residual measurement (co)variances) is a far more involved endeavor (see [6]).

In sum, it is clear that testing and adjusting for MI is critical to establishing valid inferences about group and developmental differences. However, traditional approaches to MI testing have some weaknesses. Indeed, these weaknesses are particularly problematic for longitudinal studies, in which measurement non-invariance may exist simultaneously between groups (e.g., diagnostic group, sex) and time [13].

Fortunately, the last several years have seen a wealth of psychometric innovation in the detection and adjustment of MI (e.g., Approximate Measurement Invariance and Alignment [4, 40, 41]; random effects [41]; see Davidov, Muthén, and Schmidt [14] for review). In the present study, we leverage a novel methodological approach to MI testing developed by Curran and Bauer [6, 13] that is particularly well-suited for accelerated longitudinal study designs—moderated nonlinear factor analysis (MNLFA). Conceptually, MNLFA combines the best aspects of MGA and MIMIC into a single approach. Like MGA, MNLFA allows one to adjust for non-invariance across all parameters of typical psychometric interest. However, like MIMIC models, MNLFA allows one to readily extend these tests to multiple, simultaneous moderators—either continuous or categorical.

### The present study

The present study seeks to characterize longitudinal change in RRBs in typically developing toddlers from 8 to 36 months. Distinguishing between meaningful change and measurement bias is a critical first step before modeling change, especially given the challenges associated with distinguishing between typical and atypical early RRBs. In light of these challenges, we (a) leveraged recent advances in latent variable modeling to establish a longitudinally invariant measure of RRB, and (b) used these measures to model normative and individual difference in RRB across infancy and early childhood. By modeling factor score estimates derived from MNLFA and accounting for measurement bias, the observed longitudinal trajectories will reflect what is meaningful change in behavior, as opposed to change in how the instrument measures behaviors.

## Methods

RBS-EC responses were pooled from two longitudinal studies on social development. Criteria for study participation were identical for both studies. Infants were required to (1) have no significant medical, genetic, or neurological conditions, (2) have a birthweight > 2000 g, (3) a gestational age ≥ 37 weeks, (4) have no first-degree relatives with intellectual disability, psychosis and/or schizophrenia, bipolar disorder, or autism spectrum disorder (ASD), and (5) a caregiver able to communicate in English at a level to provide informed consent. For each study, parents of infants and toddlers recruited from the University of Minnesota Institute of Child Development participant registry were invited to participate in a study about their child’s development. The registry largely reflects the racial/ethnic proportions of the broader Minneapolis—St. Paul Metropolitan area but under-represents the socio-economic diversity of this region. Parents of all participants provided informed consent and permission for their child to participate in this research study. All studies involved online questionnaires, as well as in-person visits to the lab.

In total, 612 RBS-EC questionnaires were collected from 181 participants. After excluding questionnaires with 50% or more missing data (*n* = 3), and samples taken outside of our age range (*n* = 3) our final sample comprised 606 RBS-EC questionnaires from 180 participants (88 male). Descriptive information on the final sample can be found in Table 1.

**Table 1:** Descriptive information on study sample. Ages are reported in months

Cohort	N participants	N sessions	Sessions/participant Mean (SD) [Range]	Age at first visit Mean (SD) [Range]	Average age of participants Mean (SD) [Range]
A	110 (52 female)	424	3.85 (1.5) [1,7]	11.0 (3.8) [7.4, 32.1]	17.2 (3.9) [ 9, 35.3]
B	70 (40 female)	182	2.6 (0.8) [1,3]	13.8 (3.2) [8.8, 19.3]	18.5 (1.2) [14.4, 22.1]
Combined	180 (92 female)	606	3.37 (1.4) [1,7]	12.12 (3.8) [7.4,32.1]	17.7 (3.2) [9, 35.3]

### Repetitive behavior scale for early childhood

The Repetitive Behavior Scale for Early Childhood (RBS-EC [56]) is a 34-item parent-report questionnaire that is a downward extension of the Repetitive Behavior Scale-Revised (RBS-R [7]), with good-to-excellent psychometric properties and evidence of validity and reliability (based on an independent sample of toddlers). The questionnaire is intended to capture normative variation in young children that spans across the typical-to-atypical continuum and has been used to detect difference in RRBs as a function of birthweight percentile [46], and to detect associations between RRBs and dysregulation and internalizing symptoms [30] in toddlers. For distributions of RBS-EC scores in the present sample, see Figure S1 in the Online Supplement. Each item contributes to 2 measures: items endorsed (binary) and frequency score (0-behavior does not occur, 1-behavior occurs about weekly or less, 2-behavior occurs several times a week, 3-behavior occurs about daily, and 4-behavior occurs many times a day). These measures can be summed into an overall composite measure (scored 0-34) or disaggregated into 4 psychometrically validated subscale scores: repetitive motor (scored 0-9), ritual and routine (scored 0-10), restricted behavior (scored 0-8), and self-directed behavior (scored 0-7). See http://www.cehd.umn.edu/edpsych/research/resources/rbs-ec/ for access to the instrument.

### Analytic plan

#### Factor structure

Visual inspection of distributions of item frequency responses (ranging from 0-4) indicated that responses were highly skewed—typically captured by 2 values of 0-4 scale. We therefore collapsed across empty cells by converting each item response to a binary scale based on median splits (scores ≤ the median were coded as 0, scores > the median were coded as 1). Descriptive statistics on item responses can be found in Supporting information (Table S1).

To establish configural longitudinal invariance, we first fit CFAs to two discretized age bins split at 17 months (mean age of younger group = 12.1 months (2.4), mean age of older group = 22.6 months (4.9)) (i.e., [6]) to confirm that there were separable unitary factors that items from the RBS-EC loaded on to. Repetitive behavior is not typically characterized as a singular, encapsulated construct. Rather, any meaningful characterization of repetitive behavior acknowledges that there are at least two factors that define/represent this category of behavior (e.g., lower-order and higher-order RRBs, see [51, 52]) and depending on the sample, measurement/assessment, and analytic decisions, there may be more [7, 28, 34, 37, 38, 48]. Informed by prior validation work with a sample of 17- to 27-month-olds [56], we fitted a model comprising four correlated latent factors tapping: repetitive motor, ritual and routines, restricted interests, and self-directed behavior. Items on the repetitive motor latent factor measure repetitive and non-social motor stereotypies; items on the self-directed latent factor measure repeated movement directed toward the body, including self-injury and proto-self-injury; items on the ritual and routine measure resistance to change and insistence on sameness; and items on the restricted interests measure intense or unusual interests or activities (see Online Supplement for a complete list of items). Although model fit was reasonable for the 4-factor model for both age groups (group 1: *Χ*^2^ = 542, *p* = 0.0036, RMSEA = 0.026, CFI = 0.979; group 2: *Χ*^2^ = 512, *p* = 0.04; RMSEA = 0.019; CFI = 0.991), there was an indication that two of the factors—restricted interests and rituals and routines—were so highly correlated (group 1 *φ* = 0.872, group 2 *φ* = 0.777) that they were functionally inseparable. As such, we collapsed these subscales into a single factor called “Higher-order,” where indicator residual covariances were freely estimated. Both CFAs showed good model fit (group 1 *Χ*^2^ = 572, *p* < 0.001, RMSEA = 0.029, CFI = 0.972; group 2 *Χ*^2^ = 572, *p* < 0.001, RMSEA = 0.027, CFI = 0.981).

#### Moderated nonlinear factor analysis

We used MNLFA to determine whether the RBS-EC has MI and to statistically account for any non-invariance. We were mainly concerned with longitudinal MI (i.e., whether the measurement is invariant across different ages), and used MNLFA to simultaneously determine how age as a continuous variable impacts RBS-EC latent scores, as well as its impact on DIF. Because of our two-cohort design, we also tested moderating effects of cohort, and the interaction between cohort and age since the interpretation of a main effect of age rests on the assumption that slopes do not differ between the two cohorts. Finally, we tested for moderating effects of sex, given past findings on sex-related differences in RRBs [56]. Specifically, we conducted our analyses using a slightly modified version of Gottfredson and collegues’ [21] *aMNLFA* package for R. The aMNLFA package functions as a visualization and parameterization pipeline that works with the Mplus [42] statistical program to iteratively estimate the models we detail below.

For longitudinal data, MNLFA first estimates the model parameters on a calibration sample drawn from independent observations (*n* = 180). All significant moderators on the mean and variance of the latent construct (*η*) and on DIF are combined into a final model, which is then used to estimate factor scores for the full longitudinal sample. All models were run on two independent calibration samples to test for model stability. Independent observations were pseudo-randomly selected, while attempting to ensure a similar age distribution to the full longitudinal sample (sample 1 mean age = 18.07, SD = 6.37, sample 2 mean age = 17.66 months, SD = 6.08), resulting in a 36% overlap between the two calibration samples.

Each of the three latent subscales of the RBS-EC (repetitive motor, self-directed, and higher-order RRBs) were modeled separately. First, we modeled the effect of moderators (age, sex, cohort, and age×cohort) on the latent construct (*η*). Only age was tested as a moderator on the latent variance estimates, as suggested by past literature [13]. For this initial step, model parameters with *p* < 0.1 were retained. Next, we modeled the effect of moderators on DIF (e.g., measurement artifact introduced by predictors) for each indicator intercept and loading, in the presence of moderators on the latent factor mean.

Based on the results of the initial impacts and DIF model, model parameters were then trimmed such that all effects (moderator effects on the latent mean and variance, and on DIF) with *p* < 0.05 were retained. The surviving impact and DIF effects were then tested simultaneously in one model. At this stage, Benjamini-Hochberg family-wise error correction was applied to all model parameters to protect against type I errors. Lastly, a final model was estimated using the parameters that survived the Benjamini-Hochberg correction. That model was then used to estimate the factor score estimates in the full longitudinal sample (*n* = 606), yielding person- and visit-specific estimates of *η* for each latent subscale which reflect the weighted estimates of each individual’s latent score, as well as the effects of significant moderators on DIF and *η* mean and variance.

#### Longitudinal analyses

MNLFA provides individual factor score estimates for each RBS-EC subscale adjusted for individual differences in moderating factors such as age. While MNLFA provides estimates of moderator effects on *η*, using these scores as outcomes in a multi-level model allows for the estimation of both between- and within-person effects of age. To test normative and individual differences in children’s RRB growth rates, we fitted taxonomies of growth models to each of the respective RRB subscales, moving systematically across linear and polynomial specifications of time. We subsequently added sex and cohort to model, to test for interactions with RRB growth rates. Any non-significant effects were dropped from the final model. Complete information on model comparisons for all subscales can be found in Tables 4, 5, and 6. Model comparisons were conducted using chi-square log-likelihood ratio tests and second-order Akaike Information Criteria (accounting for sample size and model complexity). All models were fitted using the lme4 and LmerTest package [5] in R 3.31.

For descriptive purposes, we fitted these models using both the MNLFA-derived factor scores and the raw-mean scores. Results for raw-mean scores can be found in the Online Supplement (Tables S2-S4). However, it should be noted that because these variables are on different scales, absolute quantitative comparisons are impossible. Indeed, the different scales between the MNLFA-derived factor scores (i.e., interval scaled) and the raw-mean scores (i.e., proportion scale) required different modeling approaches—general linear mixed models versus generalized mixed models (logistic link), respectively.

## Results

### MNLFA results

The final MNLFA model results on the structural relation between moderators and latent factor means and variances can be found in Table 2, and the final results on DIF can be found in Table 3. Overall, age was the only significant moderator of these structural models, and of item functioning. Items associated with motor ability (e.g., *lines up or arranges toys or other objects*) tended to be endorsed more frequently as infants got older, while items such as “mouths, bites, licks, or sucks objects” were endorsed less frequently with age, reflecting changes in the developmental appropriateness of such behaviors*.* Of note, higher-order items that index intense focus with objects (e.g., *focuses on parts of objects rather than the whole object*) were also endorsed less frequently with age.

**Table 2:** Results from final MNLFA model testing covariate effects on factor mean and variance

Covariate Effect	Estimate (SE)	t	*p*
**Repetitive-Motor**			
*Factor Mean*			
Age	-0.107 (0.017)	-6.334	0.000
*Factor Variance*			
Age	0.068 (0.023)	2.908	0.004
**Self-directed**			
*Factor Mean*			
Age	-0.054 (0.016)	-3.436	0.001
	.	.	.
**Higher-order**			
*Factor Variance*			
Age	0.043 (0.019)	2.223	.026

**Table 3:** Items for which there was a significant loading DIF as a function of the covariate age

Item	Loading DIF (SE)
**Repetitive Motor**	
*Mouthing Objects - mouths, bites, licks, or sucks objects*	-0.147 (0.047)
**Higher-order**	
*Arranging – lines up or arranges toys or other objects*	0.214 (0.043)
*Placement of objects – insists that things remain in the same place*	0.152 (0.045)
*Restricted use of media – strongly insists on the same music, book, app, program, movie, etc.*	0.109 (.037)
*Preoccupation with parts of objects – focuses on parts of objects rather than the whole object*	-0.303 (0.074)
*Visual inspection – closely inspects objects, views toys and other objects from an unusual angle*	-0.145 (0.039)
*Fascination with movement – intense interest or preoccupation with things that move, e.g. fans, clocks)*	-0.261 (0.058)

**Fig. 2:**
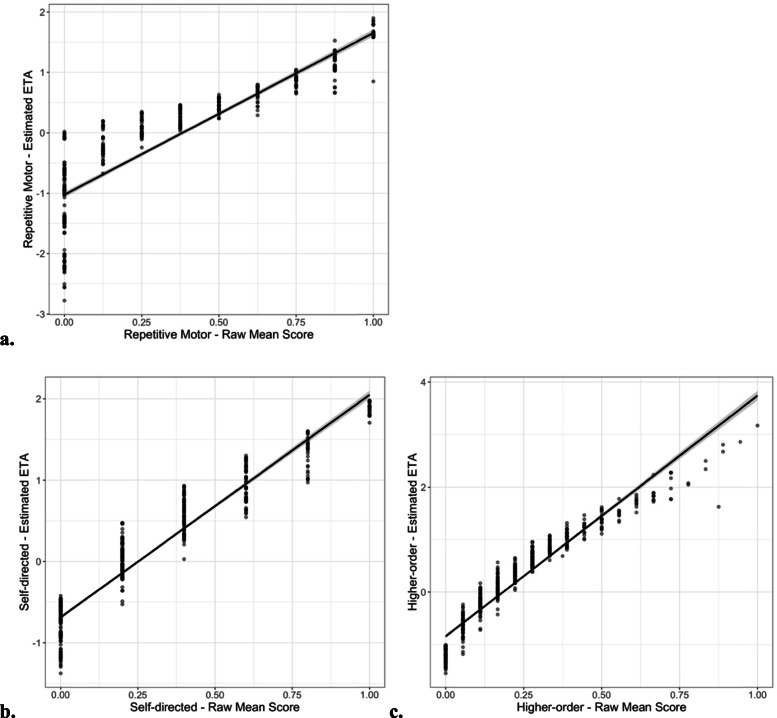
Correlation between raw mean scores and MNLFA factor scores (ETA) for (**a**) repetitive motor (*r* = 0.94), (**b**) self-directed (*r* = 0.96), and (**c**) higher-order (*r* = 0.96) behaviors (all *p*’s < 0.001)

**Fig. 3:**
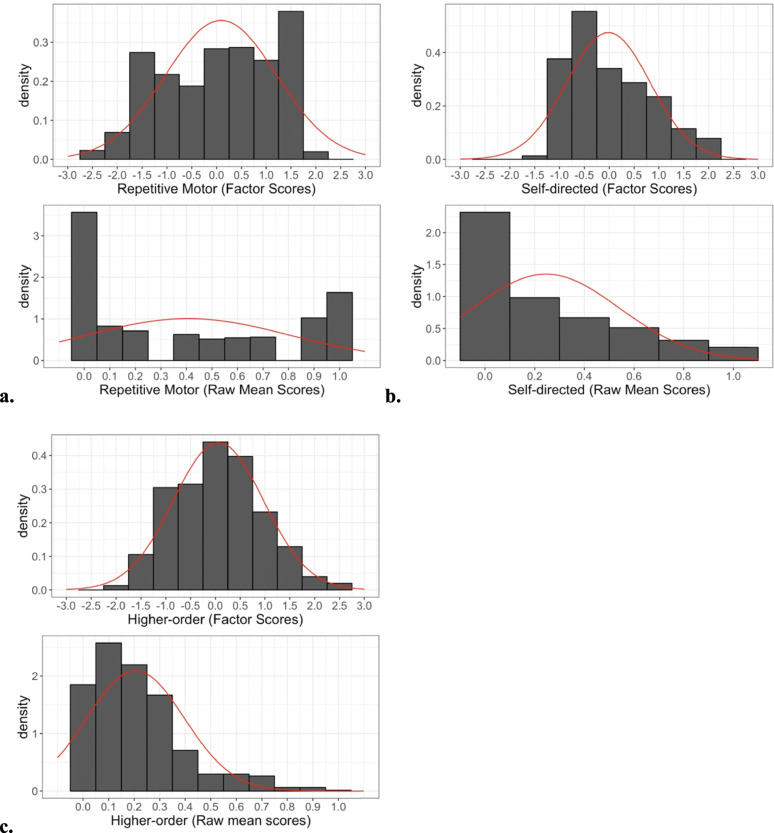
Distributions of MNLFA-derived factor scores (top) and raw-mean scores (bottom) for (**a**) repetitive motor, (**b**) self-directed, and (**c**) higher-order behaviors

MNLFA-derived factor score estimates for the longitudinal sample approached unity across the two separate calibration samples used for all three subscales (repetitive motor *r* = 0.99, self-directed *r* = 0.99, and higher-order *r* = 0.98, all *p*’s < 0.001), demonstrating that factor score estimates were not dependent upon the calibration sample used. MNLFA-derived factor scores were highly correlated with raw mean scores for all three subscales (repetitive motor (*r* = 0.93), self-directed (*r* = 0.96), and higher-order (*r* = 0.96) behaviors, all *p*’s < 0.001) However, as illustrated by Figs. 2 and 3, the MNLFA-adjusted factor scores provided considerably greater variability than the unadjusted mean scores, because scores adjust for individual-level factors that influence the degree of variation [13].

### Age-related change in restricted and repetitive behaviors

#### Repetitive motor

**Fig. 4:**
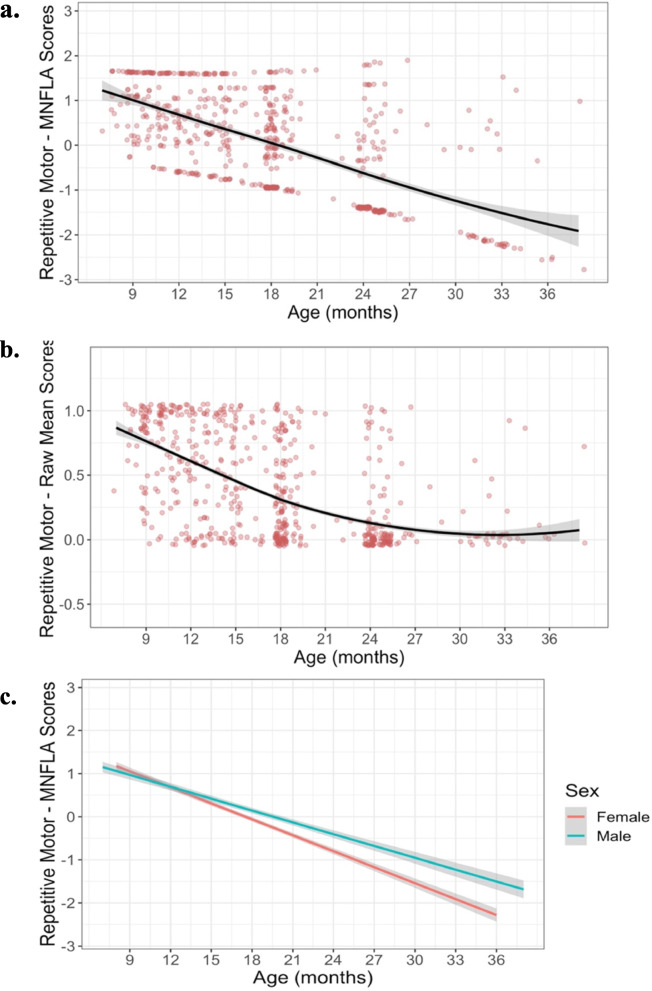
Best-fitting model estimates for Repetitive Motor scores plotted over raw data for (**a**) MNLFA-derived factor score estimates and (**b**) raw mean scores. The model outcome for the raw mean score is converted to probability, as a logistic linking function was used. *Y*-axes for both plots are scaled with limits of mean outcome +/− 2.5 SDs. **c** Impact of sex on intercept and slope. Parents of females reported fewer repetitive motor behaviors at 18 months, and their rate of decline was more rapid relative to males

Likelihood ratio tests of nested models and AIC comparisons indicated that the longitudinal MNLFA-derived factor scores for repetitive motor behavior were best represented by a linear growth function (Fig. 4). On average, children showed linear declines in their repetitive motor behaviors (*Β*_*Age*_ = −0.11, *p* < 0.0001) between 8 and 37 months of age. Scaling on the within-person variance, this corresponds to an approximate 3.0 SD decrease across this span. Notably, a statistically significant random linear slope also indicated noteworthy individual differences in children’s growth rates, around this mean trajectory (Δ−2ll = 19.1, Δdf = 2, *p* < 0.0001). Subsequent models indicated that the cohort was not predictive of differences in children’s intercepts (*B*_*Cohort*_ = 0.13, *p* = 0.2) or growth rates (*B* = −0.02, *p* = 0.13), so it was not included in the final model. Sex was predictive of differences in children’s intercepts (*B*_*sex*_ = 0.21, *p* = 0.04), and of growth rates (*B* = −0.034, *p* = 0.001) (Fig. 4); girls had slightly lower repetitive motor behaviors at 18 months and showed a more rapid decline in these behaviors than did boys. Collectively, the final model including linear age, sex, and an age×sex interaction accounted for approximately 39% of the variance in repetitive motor behavior (*R*^2^ = 0.398%). See Table 4 for full model results.

**Table 4:**
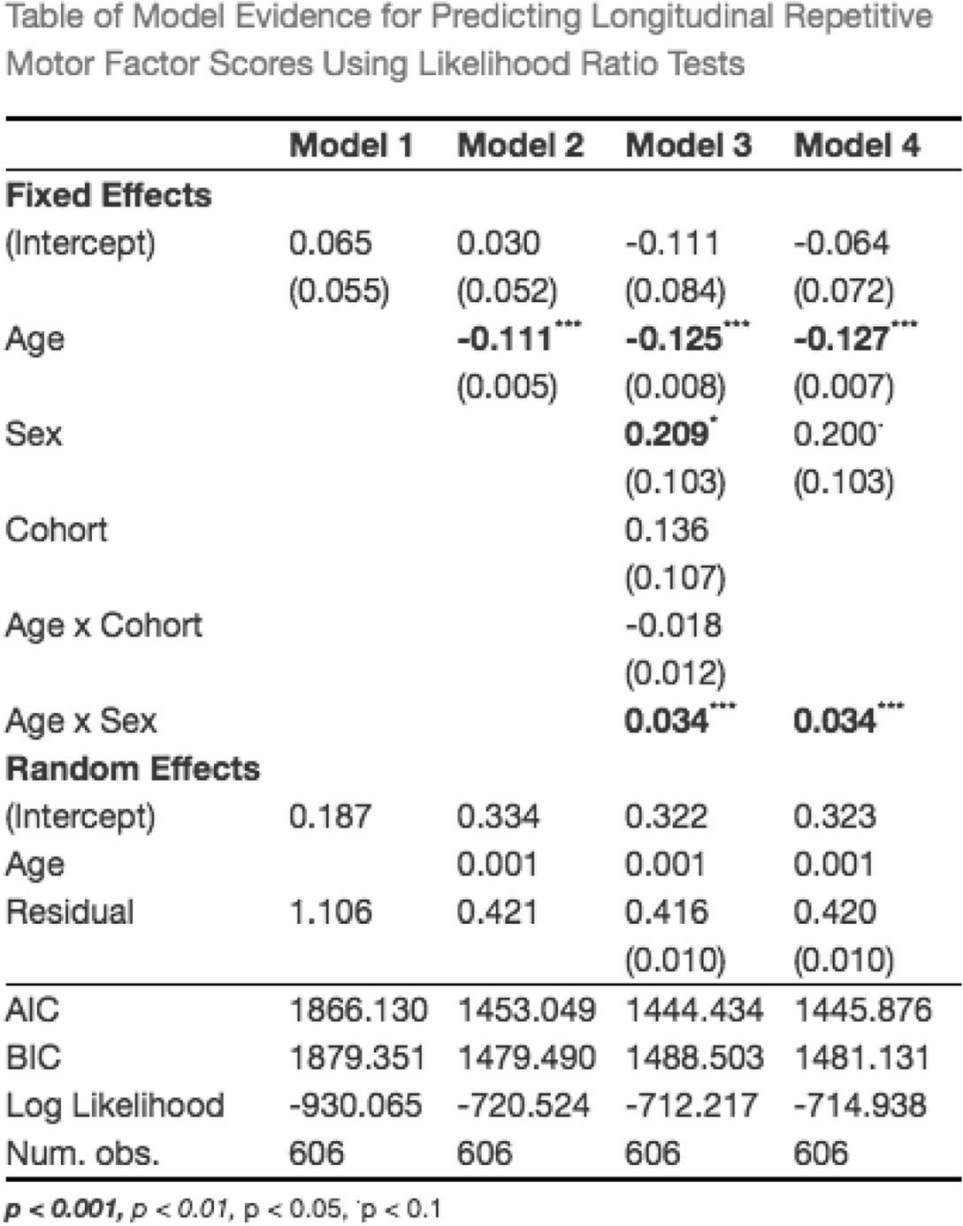
Table of model evidence for predicting repetitive motor MNLFA scores. The best-fitting model (model 4) included linear fixed and random effects of slope (age), as well as a significant effect of sex of on the intercept and slope

#### Self-directed

**Fig. 5:**
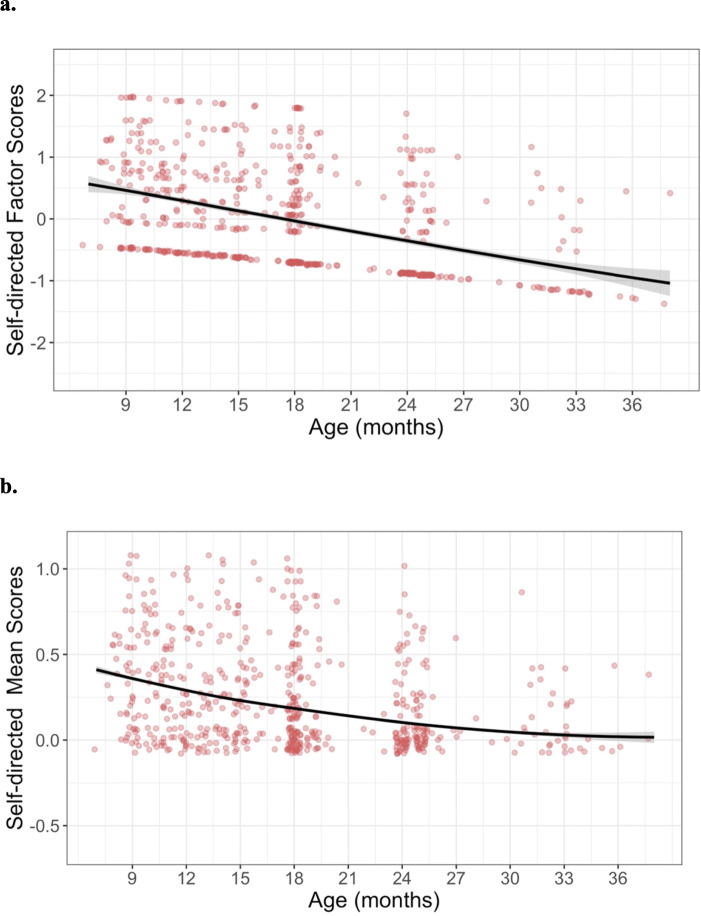
Best-fitting model estimates for self-directed scores plotted over raw data for (**a**) MNLFA-derived factor score estimates and (**b**) raw mean scores. The model outcome for the raw mean score is converted to probability, as a logistic linking function was used. *Y*-axes for both plots are scaled with limits of mean outcome +/− 3 SDs

MNLFA-derived factor scores for self-directed behaviors were best represented by a linear growth function (Fig. 5). On average, children showed linear declines in their self-directed behaviors (*Β*_*Age*_ = −0.06, *p* < 0.0001) between 8 and 37 months of age. Scaling on the within-person variance, this corresponds to an approximate 2.14 SD decrease across this span. Neither cohort nor sex was predictive of differences in children’s intercepts (*B*_*Cohort*_ = 0.07, *p* = 0.4; *B*_sex_ = 0.015, *p* = 0.86) or growth rates (*B*_*Age×Cohort*_ = −0.007, *p* = 0.5; *B*_*Age×Sex*_ = 0.009, *p* = 0.27). Collectively, the final model including linear time accounted for approximately 17.6% of the variance in self-directed behavior (*R*^2^ = 0.176). See Table 5 for full model results.

**Table 5:**
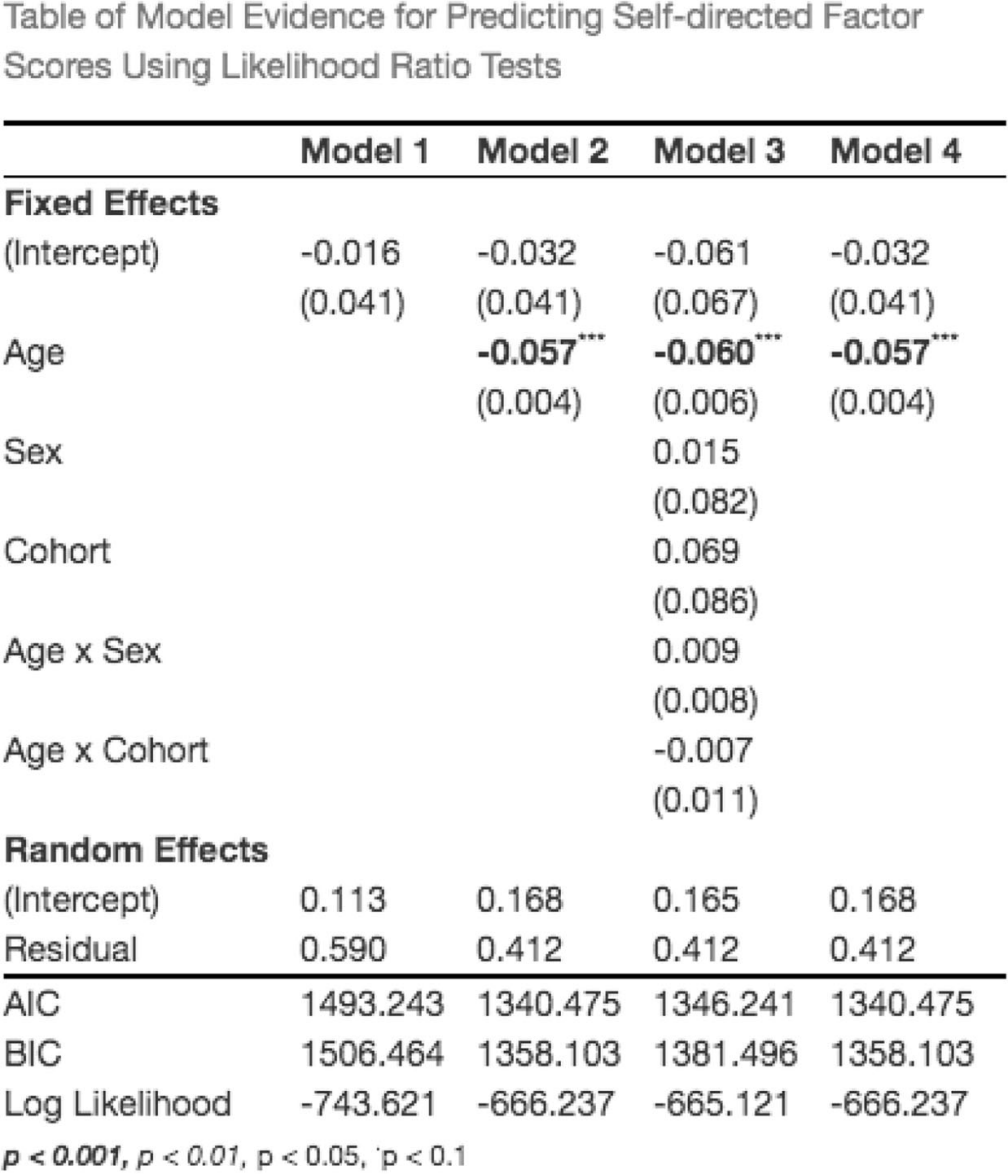
Table of model evidence for predicting self-directed MNLFA scores. The best-fitting model (model 4) included a linear fixed effect of slope (age). Neither sex nor cohort were included in the final model

#### Higher-order repetitive behavior

**Fig. 6:**
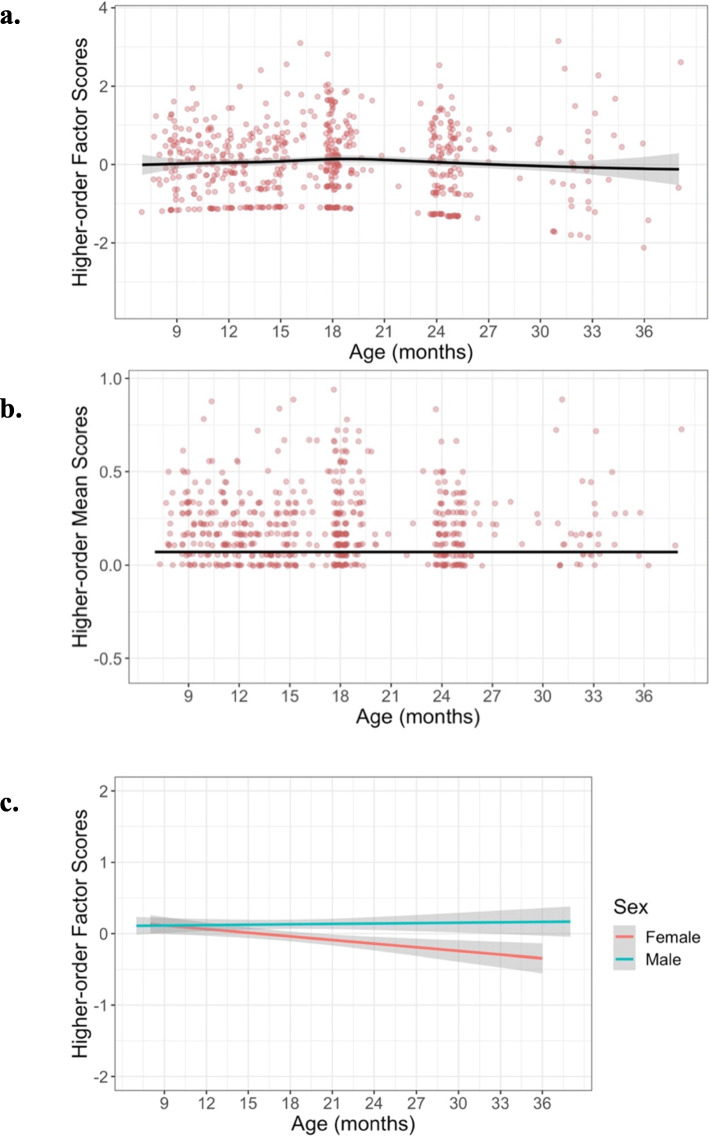
Best-fitting model estimates for higher-order scores plotted over raw data for (**a**) MNLFA-derived factor score estimates and (**b**) raw mean scores. The model outcome for the raw mean score is converted to probability, as a logistic linking function was used. *Y*-axes for both plots are scaled with limits of mean outcome +/− 4 SDs. **c** Effect of sex on slope. The decline in higher-order scores was driven by females

The longitudinal MNLFA-derived factor scores for higher-order behaviors were best represented by a quadratic growth function (Fig. 6). On average, children showed a slight decline in their higher-order behaviors at around 18 months (*B*_age_ = −0.002, *p* = 0.7; *B*_age2_ = −0.002 *p* = 0.0001). Scaling on within-person variance, this corresponds to a 0.06 SD decrease from 18 to 37 months. A statistically significant random linear slope also indicated noteworthy individual differences in children’s growth rates, around this mean trajectory (*Δ−2ll* = 11.46, *Δdf* = 2, *p* < 0.0001). Subsequent models indicated that cohort was not predictive of differences in children’s intercepts (*B*_*cohort*_ = 0.08, *p* = 0.5) or growth rates (*B* = 0.007, *p* = 0.54), so it was not included in the final model. Sex was not predictive of differences in children’s intercepts (*B =* 0.19, *p* = 0.08), but was predictive of linear growth rates (*B =* 0.024, *p* = 0.01) (Fig. 6); while boys and girls had similar higher-order behaviors at 18 months, girls showed a slight decline in these behaviors, while boys stayed flat. Collectively, the final model including linear and quadratic age, sex and an age×sex interaction accounted for approximately 74% of the variance in higher-order behaviors (*R*^2^ = 0.74). See Table 6 for full model results.

**Table 6:**
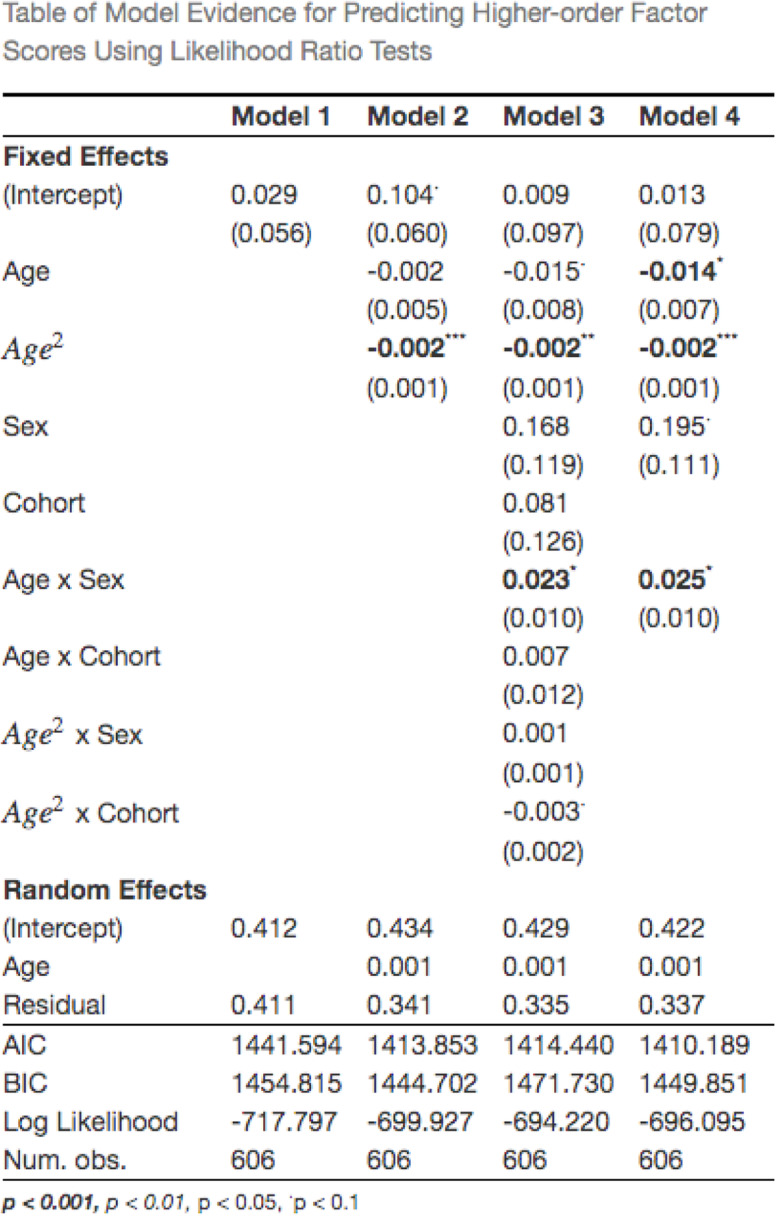
Table of model evidence for predicting higher-order MNLFA scores. The best-fitting model (model 4) included a quadratic fixed effect and linear random effect for slope (age), and a significant effect of sex on intercept and slope

### Value added

What if we had simply used the unadjusted mean scores, as opposed to the MNLFA-adjusted factor scores? As noted, the correlations between the unadjusted mean scores and MNLFA-adjusted factor scores were very strong. However, failing to account for longitudinal non-invariance led to some non-trivial differences in longitudinal trajectories. Most notably, when using raw unadjusted scores the observed decline in repetitive motor (Fig. 4) and self-directed behaviors (Fig. 5) flattened out over time (full model results can be found in the Online supplement). This may be because modeling the bounded and right-skewed raw-mean data for these subscale results in a floor-effect for the older toddlers. Further issues arise when modeling raw unadjusted scores that afford limited variance. For example, for the repetitive motor subscale, the model failed to converge when we attempted to test for the effect of sex and cohort on intercept and growth rate, suggesting that with the limited variance afforded by proportion scores, only a limited number of parameters could be included in the model. For the self-directed subscale, non-significant random effects for the instantaneous linear slopes indicated that all children showed statistically identical growth rates.

Similarly, failing to account for longitudinal non-invariance and adopting the bounded and right-skewed raw-mean higher-order behavior scale led to some non-trivial differences in children’s higher-order growth trajectories, as compared with invariance-adjusted MNLFA scores (Fig. 6). In this case, the best-fitting model for the raw mean scores included only fixed and random effects of intercept, and we were unable to detect either within- or between-person growth effects.

## Discussion

The goal of the present study was to characterize normative rates of RRBs and their subsequent change over time in a large longitudinal community sample of toddlers. We tested longitudinal MI in the RBS-EC in order to examine the extent to which our constructs (repetitive motor, self-directed, and higher-order behaviors) were commensurate across child demographic covariates (age and sex). We found no differences in the meaning of scale (i.e., non-invariance) due to sex. However, there were some differences as a function of age, with the higher-order subscale showing the most evidence of DIF. Interestingly, many of these items describe unusual objects and visual exploration (e.g., *focuses on parts of objects rather than the whole objects*, *closely inspects objects*, *lines up or arranges toys).* Past work has shown that unusual object and visual exploration at 12-months is associated with subsequent autism severity scores [43], suggesting that these items may be useful for predicting emerging atypicalities during a specific developmental window. However, past work has also demonstrated low rates of similar behaviors in 2-year-olds with ASD and non-spectrum developmental disorders [45], suggesting that they may be challenging to measure in the first years of life regardless of diagnostic group. Thus, future work comparing the trajectories of these behaviors between ASD and TD children must be careful to adjust for DIF both as a function of age and diagnostic status. Crucially, MNLFA scores adjust for DIF and therefore generate more precise estimates of these constructs.

Our model results indicate that RRB subscales have differing developmental trajectories, which in part validates their utility as separable constructs [7, 33, 51]. Self-directed behaviors showed a linear decrease from 8 to 36 months, as did repetitive motor behaviors which showed a slower decline in boys relative to girls. These findings corroborate past longitudinal work showing a decline in repetitive and sensory-motor behaviors beginning at 15 months, as well as higher rates of these behaviors in boys beginning at 15 months that becomes statistically higher than girls by 77 months [53]. We found that higher-order behaviors begin to decline later in development relative to lower-order behaviors, also corroborating past cross-sectional [18] and longitudinal work [53].

Of note, our work is the first to longitudinally measure RRBs beginning in the first year of life. Past work has raised the possibility of using parent reports of RRBs to augment early screening for ASD [55], and characterizing these early behavioral trajectories in typically developing children is a critical first step toward understanding when and how they deviate from their ASD counterparts. By using the RBS-EC, we were able to measure age-related declines in self-directed behaviors, which may prove especially important in distinguishing between clinical and developmentally normative presentations of RRBs.

## Conclusions

Why use MNLFA scores to examine rates of RRBs, as opposed to raw mean scores? First, adjusting for DIF due to individual differences in age provided us with more within-person variability to model within-person changes in RRBs. This has potential implications for measuring behavioral change over time in treatment and intervention studies, beyond RRBs. Many have argued for the importance of outcome measures that are sensitive enough to capture subtle within-person change for assessing treatment outcomes (e.g., [27]). We argue in addition to the sensitivity of the measure itself, using scores that adjust for DIF confers the additional benefit of increasing within-person variability when measuring individual treatment outcomes or efficacy.

Second, these scores disentangle measurement bias introduced by age and true changes in the latent construct over time, allowing us to more accurately estimate their developmental trajectories. This may be particularly important in follow-up work examining group differences between ASD and TD toddlers in these trajectories, as DIF due to age may interact with DIF due to risk status. For example, in the present study, we found that items associated with routinized play (e.g., *lines up or arranges toys or other objects*) tended to be endorsed more frequently as infants got older. It is likely that this item functions differently in populations with neurodevelopmental disorders, in addition to DIF due to age. When comparing behavioral trajectories in TD and ASD infants, we must have a clear understanding of the underlying factor structure and be able to statistically adjust for biases in our measures due to diagnostic group and age for this information to be interpretable. Validity and reliability are not inherent attributes of an instrument but are inextricably linked to the sample in which they were established. Indeed, establishing MI is essential in order to avoid what Meehl referred to as “detached validity claims” [35], in which instruments psychometrically verified in one sample or at one age are assumed to function similarly in other samples or ages of children. The implications of such assumptions are broad and potentially costly. Thankfully, with recent simplifications in the implementation of MNLFA [21], researchers can adjust for non-invariance more easily than ever.

Adjusting for non-invariance is critical when comparing a latent construct over time or between groups, however, we are still limited in our ability to make causal inferences. Though we identified DIF as a function of age, a third variable correlated with age such as language development could be driving these effects. For example, past work has shown that early changes in RRBs are associated with developmental factors such as language and socio-cognitive development [19, 29, 32]. Multivariate analyses using latent factor scores adjusted for non-invariance for RRBs and covariates of interest are needed to further understand the interplay of these factors across development.

In sum, this paper provides foundational evidence of the developmental trajectories of RRB sub-types among typically developing children. We found that adjusting for non-invariance as a function of age provided more accurate estimates of developmental trajectories by ensuring that factor score estimates were placed on the same developmental scale. Future work will test the invariance of these metrics in a sample enriched for ASD risk and will consider the role of language and cognition in RRBs across the typical-to-atypical continuum.

## Supplementary Information

**Additional file 1:** **Figure S1.** Sample distribution (*n*=606) for total items endorsed (Mean=8.79, SD=6.2). **Table S1.** Median item-level responses (min=0, max=4). Each item was converted to a binary scale (0, 1) using its median split. These binarized scores were then used to calculate the proportion of samples endorsing a given item. **Table S2.** Linear Mixed Effects Model results testing the effect of Age, Cohort, and Sex on Repetitive Motor raw mean scores. **Model 1**=Baseline model; **Model 2**=Establishing functional form; Linear fixed and random effects of age. Model did not converge when covariates (Cohort and Sex) were included, and was not considered as a candidate model, so **Model 2** was adopted as final model. **Table S3.** Linear Mixed Effects Model results testing the effect of Age, Cohort, and Sex on Self-directed raw mean scores. **Model 1**=Baseline model; **Model 2**=Establishing functional form; Linear fixed effects of age. **Model 3**=Assessing covariates. **Model 4=**Final model with significant covariate of Cohort included. **Table S4.** Linear Mixed Effects Model results testing the effect of Age, Cohort, and Sex on Higher-order raw mean scores. **Model 1**=Baseline model. **Model 2**=Adding covariates (initial model establishing functional form found no significant fixed or random effects of Age). **Model 3=**Final model selected after removing non-significant effects of Sex and Cohort.

## Data Availability

The datasets analyzed during the current study and the analysis scripts are available in the https://github.com/rrobinn/invariance-repetitive-bx repository.

## Abbreviations

ASD: Autism spectrum disorders
DIF: Differential item functioning
HR-ASD: Individuals at high-familial risk for ASD who later received a diagnosis
HR-Neg: Individuals at high-familial risk for ASD who do not receive a diagnosis
MGA: Multiple groups analysis
MI: Measurement invariance
MIMIC: Multiple indicators, multiple causes
MNLFA: Moderated nonlinear factor analysis
RBS-EC: Repetitive Behavior Scales for Early Childhood
RBQ-2: Repetitive Behaviors Questionnaire-2
RRBs: Restricted and repetitive behaviors
TD: Typically developing

## References

[1] American Psychiatric Association (2013). Diagnostic and statistical manual of mental disorders.

[2] American Psychological Association [APA]. APA dictionary of statistics and research methods. Washington: American Psychological Association; 2014.

[3] Arnott B, McConachie H, Meins E, Fernyhough C, Le Couteur A, Turner M (2010). The frequency of restricted and repetitive behaviors in a community sample of 15-month-old infants. J Dev Behav Pediatr.

[4] Asparouhov T, Muthén B (2014). Multiple-group factor analysis alignment. Struct Equ Model.

[5] Bates D, Mächler M, Bolker BM, Walker SC. Fitting linear mixed-effects models using lme4. J Stat Softw. 2015;67(1):1-42. 10.18637/jss.v067.i01.

[6] Bauer DJ (2017). A more general model for testing measurement invariance and differential item functioning. Psychol Models.

[7] Bodfish JW, Symons FJ, Parker DE, Lewis MH (2000). Varieties of repetitive behaviour in autism: comparison to mental retardation. J Autism Dev Disord.

[8] Byrne BM, Muthèn BO, Shavelson RJ (1989). Testing the equivalence of factor covariance and mean structure: the issue of partial measurement invariance. Psychol Bull.

[9] Byrne BM, Watkins D (2003). The issue of measurement invariance revisited. J Cross-Cult Psychol.

[10] Cicchetti D. Developmental psychopathology: reactions, relfections, projections. Dev Rev. 1993. 10.1006/drev.1993.1021.

[11] Constantino JN (2011). The quantitative nature of autistic social impairment. Pediatr Res.

[12] Constantino JN (2018). Deconstructing autism: from unitary syndrome to contributory developmental endophenotypes. Int Rev Psychiatry.

[13] Curran PJ, Mcginley JS, Bauer DJ, Andrea M, Burns A, Chassin L, Sher K (2014). A moderated nonlinear factor model for the development of commensurate measures in integrative data analysis. Multivar Behav Res.

[14] Davidov E, Muthen B, Schmidt P (2018). Measurement invariance in cross-national studies: challenging traditional approaches and evaluating new ones. Sociol Methods Res.

[15] DeLoache JS, Simcock G, Macari S (2007). Planes, trains, automobiles-and tea sets: extremely intense interests in very young children. Dev Psychol.

[16] Elison JT, Wolff JJ, Reznick JS, Botteron KN, Estes AM, Gu H (2014). Repetitive behavior in 12-month-olds later classified with autism spectrum disorder. J Am Acad Child Adolesc Psychiatry.

[17] Evans D, Gray FL, Leckman JF. The rituals, fears and phobias of young children: insights from development, psychopathology and neurobiology. Child Psychiatry Hum Dev. 1998;29(4):261-76. 10.1023/a:1021392931450 10422351

[18] Evans DW, Leckman JF, Carter A, Reznick S, Henshaw D, King RS, Pauls D (1997). Ritual, habit, and perfectionism: the prevalence and development of compulsive-like behavior in normal young children. Child Dev.

[19] Evans DW, Lewis MD, Iobst E (2004). The role of the orbito- frontal cortex in normally developing compulsive-like behaviors and obsessivecompulsive disorder. Brain Cognit.

[20] Evans DW, Uljarević M, Lusk LG, Loth E, Frazier T (2017). Development of two dimensional measures of restricted and repetitive behavior in parents and children. J Am Acad Child Adolesc Psychiatry.

[21] Gottfredson NC, Cole VT, Giordano ML, Bauer DJ, Hussong AM, Ennett ST (2018). Simplifying the implementation of modern scale scoring methods with an automated R package: automated moderated nonlinear factor analysis (aMNLFA). Addict Behav.

[22] Harrop C, McConachie H, Emsley R, Leadbitter K, Green J (2014). Restricted and repetitive behaviors in autism spectrum disorders and typical development: cross-sectional and longitudinal comparisons. J Autism Dev Disord.

[23] Hoch J, Spofford L, Dimian A, Tervo R, Maclean WE, Symons FJ (2016). A direct comparison of self-injurious and stereotyped motor behavior between preschool-aged children with and without developmental delays. J Pediatr Psychol.

[24] Honey E, Rodgers J, McConachie H (2012). Measurement of restricted and repetitive behaviour in children with autism spectrum disorder: selecting a questionnaire or interview. Res Autism Spectr Disord.

[25] Jöreskog KG (1971). Simultaneous factor analysis in several populations. Psychometrika.

[26] Jöreskog KG, Goldberger AS (1975). Estimation of a model with multiple indicators and multiple causes of a single latent variable. J Am Stat Assoc.

[27] Kim SH, Grzadzinski R, Martinez K, Lord C (2019). Measuring treatment response in children with autism spectrum disorder: applications of the brief observation of social communication change to the autism diagnostic observation schedule. Autism.

[28] Lam KSL, Bodfish JW, Piven J (2008). Evidence for three subtypes of repetitive behavior in autism that differ in familiality and association with other symptoms. J Child Psychol Psychiatry Allied Discip.

[29] Larkin F, Meins E, Centifanti L, Fernyhough C, Leekam S (2017). How does restricted and repetitive behavior relate to language and cognition in typical development?. Dev Psychopathol.

[30] Lasch C, Wolff JJ, Elison JT. Examining criterion-oriented validity of the Repetitive Behavior Scales for Early Childhood (RBS-EC) and the Video-Referenced Rating of Reciprocal Social Behavior (vrRSB). Dev Psychopathol. 2019:37(3):779-89. 10.1017/S0954579419001159. 10.1017/S0954579419001159 PMC7047542 31455435

[31] Leekam S, Tandos J, McConachie H, Meins E, Parkinson K, Wright C (2007). Repetitive behaviours in typically developing 2-year-olds. J Child Psychol Psychiatry Allied Discip.

[32] Leekam SR, Prior MR, Uljarevic M (2011). Restricted and repetitive behaviors in autism spectrum disorders: A review of research in the last decade. Psychol Bull.

[33] Lewis MH, Bodfish JW (1998). Repetitive behavior disorders in autism. Ment Retard Dev Disabil Res Rev.

[34] Mandy WPL, Skuse DH (2008). Research review: what is the association between the social-communication element of autism and repetitive interests, behaviours and activities?. J Child Psychol Psychiatry Allied Discip.

[35] Meehl PE (1990). Why summaries of research on psychological theories are often uninterpretable. Psychol Rep.

[36] Mellenbergh GJ (1989). Item bias and item response theory. Int J Educ Res.

[37] Mirenda P, Smith IM, Vaillancourt T, Georgiades S, Duku E, Szatmari P (2010). Validating the repetitive behavior scale-revised in young children with autism spectrum disorder. J Autism Dev Disord.

[38] Mooney EL, Gray KM, Tonge BJ, Sweeney DJ, Taffe JR (2009). Factor analytic study of repetitive behaviours in young children with pervasive developmental disorders. J Autism Dev Disord.

[39] Moss J, Oliver C, Arron K, Burbidge C, Berg K (2009). The prevalence and phenomenology of repetitive behavior in genetic syndromes. J Autism Dev Disord.

[40] Muthen B, Asparouhov T (2014). IRT studies of many groups: the alignment method. Front Psychol.

[41] Muthén B, Asparouhov T (2018). Recent methods for the study of measurement invariance with many groups: alignment and random effects. Sociol Methods Res.

[42] Muthén LK, Muthen B. Mplus user's guide: Statistical analysis with latent variables, user's guide. Muthén & Muthén; 2017.

[43] Ozonoff S, Macari S, Young GS, Goldring S, Thompson M, Rogers SJ (2008). Atypical object exploration at 12 months of age is associated with autism in a prospective sample. Autism.

[44] Putnick DL, Bornstein MH (2016). Measurement invariance conventions and reporting. Dev Rev.

[45] Richler J, Bishop SL, Kleinke JR, Lord C (2007). Restricted and repetitive behaviors in young children with autism spectrum disorders. J Autism Dev Disord.

[46] Sifre R, Lasch C, Fenoglio A, Georgieff MK, Wolff JJ, Elison JT (2018). Restricted, repetitive, and reciprocal social behavior in toddlers born small for gestation duration. J Pediatr.

[47] Steinberg L, Thissen D. Using effect sizes for research reporting: examples using item response theory to analyze differential item functioning. Psychol Methods. 2006. 10.1037/1082-989X.11.4.402. 10.1037/1082-989X.11.4.402 17154754

[48] Szatmari P, Georgiades S, Bryson S, Zwaigenbaum L, Roberts W, Mahoney W (2006). Investigating the structure of the restricted, repetitive behaviours and interests domain of autism. J Child Psychol Psychiatry Allied Discip.

[49] Thelen E (1979). Rhythmical stereotypies in normal human infants. Anim Behav.

[50] Thelen E (1981). Rhythmical behavior in infancy: an ethological perspective. Dev Psychol.

[51] Turner M, Russell J (1997). Towards an executive dysfunction account of repetitive behavior in autism. Autism as an executive disorder.

[52] Turner M (1999). Repetitive behaviour in autism: a review of psychological research. J Child Psychol Psychiatry.

[53] Uljarević M, Arnott B, Carrington SJ, Meins E, Fernyhough C, McConachie H (2017). Development of restricted and repetitive behaviors from 15 to 77 months: stability of two distinct subtypes?. Dev Psychol.

[54] Wolff JJ, Bodfish JW, Hazlett HC, Lightbody AA, Reiss AL, Piven J (2012). Evidence of a distinct behavioral phenotype in young boys with fragile x syndrome and autism. J Am Acad Child Adolesc Psychiatry.

[55] Wolff JJ, Botteron KN, Dager SR, Elison JT, Estes AM, Gu H (2014). Longitudinal patterns of repetitive behavior in toddlers with autism. J Child Psychol Psychiatry Allied Discip.

[56] Wolff JJ, Boyd BA, Elison JT. A quantitative measure of restricted and repetitive behaviors for early childhood. J Neurodev Disord. 2016;8(27). 10.1186/s11689-016-9161-x. 10.1186/s11689-016-9161-x PMC4970296 27486483

