# Supplementary material for: Longitudinal change in restricted and repetitive behaviors from 8-36 months
Source: J Neurodev Disord. 2021 Jan 11;13:7. doi: 10.1186/s11689-020-09335-0 (PMC7798225; doi:10.1186/s11689-020-09335-0)
Supplement: Supplementary file 1 — Additional file 1: Figure S1. Sample distribution (n=606) for total items endorsed (Mean=8.79, SD=6.2). Table S1. Median item-level responses (min=0, max=4). Each item was converted to a binary scale (0, 1) using its median split. These binarized scores were then used to calculate the proportion of samples endorsing a given item. Table S2. Linear Mixed Effects Model results testing the effect of Age, Cohort, and Sex on Repetitive Motor raw mean scores. Model 1=Baseline model; Model 2=Establishing functional form; Linear fixed and random effects of age. Model did not converge when covariates (Cohort and Sex) were included, and was not considered as a candidate model, so Model 2 was adopted as final model. Table S3. Linear Mixed Effects Model results testing the effect of Age, Cohort, and Sex on Self-directed raw mean scores. Model 1=Baseline model; Model 2=Establishing functional form; Linear fixed effects of age. Model 3=Assessing covariates. Model 4=Final model with significant covariate of Cohort included. Table S4. Linear Mixed Effects Model results testing the effect of Age, Cohort, and Sex on Higher-order raw mean scores. Model 1=Baseline model. Model 2=Adding covariates (initial model establishing functional form found no significant fixed or random effects of Age). Model 3=Final model selected after removing non-significant effects of Sex and Cohort. [file 11689_2020_9335_MOESM1_ESM.docx]

**Online Supplement**

**Linear mixed effect models**

To account for the skew in the raw-mean scores, we used generalized linear mixed models with a logistic linking function. All models were fitted using the glm function in the stats package in R 3.31. For replicability, we report the untransformed model parameter estimates in Tables S2-S4. For interpretability of effects, we converted the effects to probabilities for the visualizations in the main body of the manuscript.

| **Description** | **Median Raw Score** | **Endorsement (%)** |
| --- | --- | --- |
| *Repetitive Motor* |  |  |
| LEGS | 3 | 0.49 |
| HEAD | 2 | 0.38 |
| WHOLE BODY/TORSO | 2 | 0.45 |
| ARMS/HANDS/FINGERS | 2 | 0.41 |
| ARMS/HANDS/FINGERS ON SURFACES | 3 | 0.41 |
| OBJECT USAGE | 4 | 0.37 |
| MOUTHING OBJECTS | 4 | 0.35 |
| LOCOMOTION | 2 | 0.44 |
| VOCALIZATIONS | 2 | 0.44 |
| *Self-Directed* |  |  |
| HITS SELF WITH BODY PART | 1 | 0.27 |
| HITS SELF WITH OBJECT | 1 | 0.22 |
| HITS SELF AGAINST SURFACE | 1 | 0.27 |
| RUBS, SCRATCHES, POKES OR PINCHES SELF | 1 | 0.2 |
| PULLS OWN HAIR | 1 | 0.26 |
| *Restricted Interests/Ritual & Routine* |  |  |
| LIMITED & INTENSE INTERESTS | 1 | 0.28 |
| RESTRICTED USE OF MEDIA | 1 | 0.22 |
| SENSORY INTERESTS | 1 | 0.24 |
| STRONGLY ATTACHED TO SPECIFIC OBJECT | 1 | 0.35 |
| VOCALIZATIONS | 1 | 0.23 |
| STILLNESS | 1 | 0.10 |
| VISUAL INSPECTION | 1 | 0.41 |
| FASCINATION WITH MOVEMENT | 1 | 0.45 |
| ARRANGING | 1 | 0.27 |
| PLACEMENT OF OBJECTS | 1 | 0.15 |
| UPSET VISITING NEW PLACES | 1 | 0.12 |
| UPSET IF INTERRUPTED | 1 | 0.35 |
| APPEARANCE OF OTHERS | 1 | 0.10 |
| INFLEXIBLE ROUTINE | 1 | 0.08 |
| EATING/MEALTIME | 1 | 0.11 |
| SLEEPING/BEDTIME | 1 | 0.15 |
| PLAY | 1 | 0.04 |
| SOCIAL INTERACTION | 1 | 0.03 |

*Table S1*. Median item-level responses (min=0, max=4). Each item was converted to a binary scale (0,1) using its median split. These binarized scores were then used to calculate the proportion of samples endorsing a given item.


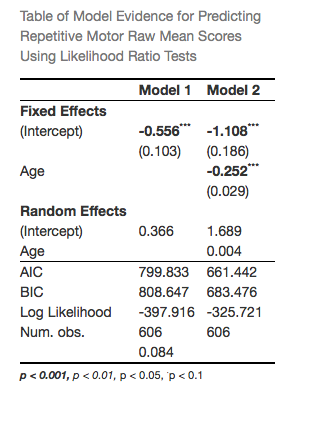


Table S2. Linear Mixed Effects Model results testing the effect of Age, Cohort, and Sex on Repetitive Motor raw mean scores. **Model 1**=Baseline model; **Model 2**=Establishing functional form; Linear fixed and random effects of age. Model did not converge when covariates (Cohort and Sex) were included, and was not considered as a candidate model, so **Model 2** was adopted as final model.


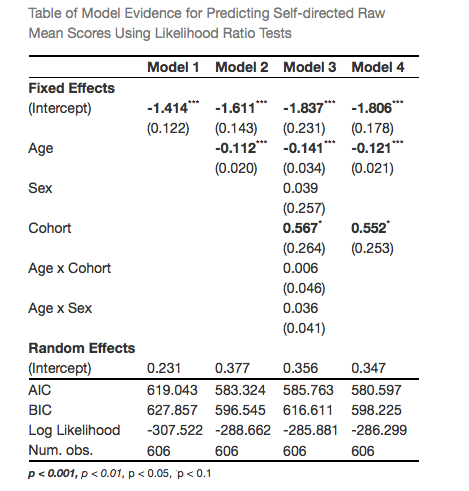


Table S3. Linear Mixed Effects Model results testing the effect of Age, Cohort, and Sex on Self-directed raw mean scores. **Model 1**=Baseline model; **Model 2**=Establishing functional form; Linear fixed effects of age. **Model 3**=Assessing covariates. **Model 4=**Final model with significant covariate of Cohort included.


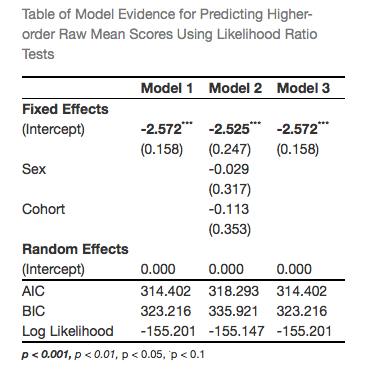


Table S4. Linear Mixed Effects Model results testing the effect of Age, Cohort, and Sex on Higher-order raw mean scores. **Model 1**=Baseline model. **Model 2**=Adding covariates (initial model establishing functional form found no significant fixed or random effects of Age). **Model 3=**Final model selected after removing non-significant effects of Sex and Cohort.

##
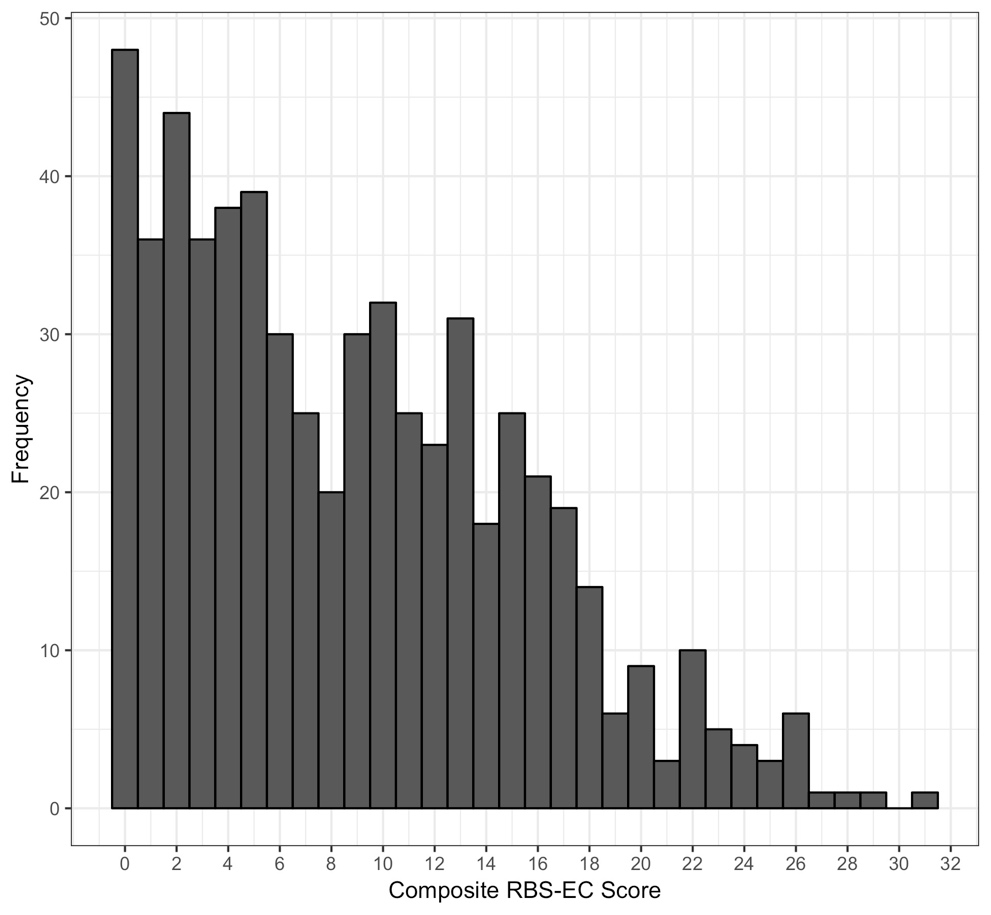


Figure S1. Sample distribution (n=606) for total items endorsed (Mean=8.79, SD=6.2).
